# (4-But­oxy­phen­yl)(1*H*-pyrrol-2-yl)methanone

**DOI:** 10.1107/S1600536812017370

**Published:** 2012-04-25

**Authors:** V. Prakash, Kamini Kapoor, M. Shet Prakash, Vivek K. Gupta, Rajni Kant

**Affiliations:** a Shirdi Sai Engineering College, Anekal, Bangalore 562 106, India; b X-ray Crystallography Laboratory, Post-Graduate Department of Physics & Electronics, University of Jammu, Jammu Tawi 180 006, India; c Centre for Advanced Materials, Tumkur University, Tumkur, India; d Department of Chemistry, University College of Science, Tumkur University, Tumkur, India, Tumkur, India

## Abstract

The asymmetric unit of the title compound, C_15_H_17_NO_2_, contains two independent mol­ecules in which the dihedral angles between the pyrrole and benzene rings are 42.43 (9) and 45.70 (9)°. In both mol­ecules, the but­oxy chains are disordered over two sets of sites, with occupancy ratios of 0.701 (7):0.299 (7) and 0.869 (4):0.131 (4). Each mol­ecule forms a dimer with an inversion-related mol­ecule, through a pair of N—H⋯O hydrogen bonds. Weak C—H⋯O inter­actions link these dimers in the crystal structure.

## Related literature

For background and applications of pyrrole derivatives, see: Fischer & Orth (1934 ▶); Mohamed *et al.* (2009 ▶). For related structures, see: English *et al.* (1980 ▶).
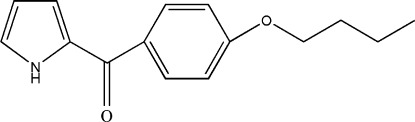


## Experimental

### 

#### Crystal data

C_15_H_17_NO_2_
*M* *_r_* = 243.30
Triclinic, 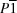

*a* = 9.4779 (4) Å
*b* = 11.4478 (5) Å
*c* = 13.1117 (7) Å
α = 95.155 (4)°
β = 104.118 (4)°
γ = 94.626 (3)°
*V* = 1366.31 (11) Å^3^
*Z* = 4
Mo *K*α radiation
μ = 0.08 mm^−1^
*T* = 293 K
0.3 × 0.2 × 0.2 mm

#### Data collection

Oxford Diffraction Xcalibur Sapphire3 diffractometer
Absorption correction: multi-scan (*CrysAlis RED*; Oxford Diffraction, 2010 ▶) *T*_min_ = 0.935, *T*_max_ = 1.000
13503 measured reflections
5344 independent reflections
3273 reflections with *I* > 2σ(*I*)
*R*_int_ = 0.028

#### Refinement

*R*[*F* ^2^ > 2σ(*F* ^2^)] = 0.056
*wR*(*F* ^2^) = 0.160
*S* = 1.03
5344 reflections
385 parameters
38 restraints
H atoms treated by a mixture of independent and constrained refinement
Δρ_max_ = 0.32 e Å^−3^
Δρ_min_ = −0.18 e Å^−3^

### 

Data collection: *CrysAlis PRO* (Oxford Diffraction, 2010 ▶); cell refinement: *CrysAlis PRO*; data reduction: *CrysAlis RED* (Oxford Diffraction, 2010 ▶); program(s) used to solve structure: *SHELXS97* (Sheldrick, 2008 ▶); program(s) used to refine structure: *SHELXL97* (Sheldrick, 2008 ▶); molecular graphics: *ORTEP-3* (Farrugia, 1997 ▶); software used to prepare material for publication: *PLATON* (Spek, 2009 ▶).

## Supplementary Material

Crystal structure: contains datablock(s) I, global. DOI: 10.1107/S1600536812017370/bh2427sup1.cif

Structure factors: contains datablock(s) I. DOI: 10.1107/S1600536812017370/bh2427Isup2.hkl

Supplementary material file. DOI: 10.1107/S1600536812017370/bh2427Isup3.cml

Additional supplementary materials:  crystallographic information; 3D view; checkCIF report

## Figures and Tables

**Table 1:** Hydrogen-bond geometry (Å, °)

*D*—H⋯*A*	*D*—H	H⋯*A*	*D*⋯*A*	*D*—H⋯*A*
N1*A*—H1*A*⋯O7*A*^i^	0.93 (3)	2.00 (2)	2.864 (2)	154 (2)
N1*B*—H1*B*⋯O7*B*^ii^	0.88 (3)	2.06 (2)	2.834 (3)	146 (2)
C9*B*—H9*B*⋯O7*A*^i^	0.93	2.59	3.426 (3)	149

Symmetry codes: (i) 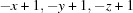; (ii) 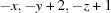.

**Fig. 1.**
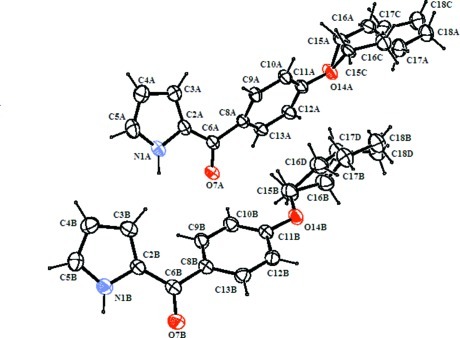
ORTEP view of the title compound with thermal ellipsoids drawn at the 40% probability level. H atoms are shown as small spheres of arbitrary radii.

**Fig. 2.**
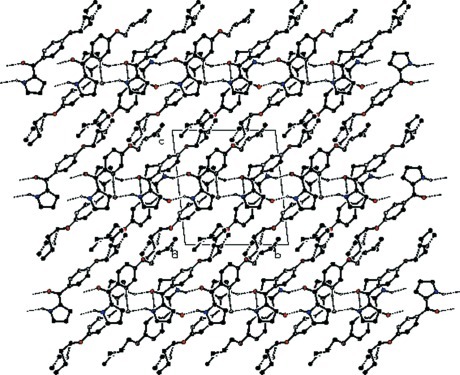
A molecular packing view of the title compound, showing intermolecular interactions. For clarity, hydrogen atoms not involved in hydrogen bonding have been omitted.

## supplementary crystallographic information

### Comment

The chemistry of pyrrole compounds and biological activities of the related compounds have been extensively studied (Fischer & Orth, 1934; Mohamed *et al.*, 2009). With the view of biological importance, the title compound was synthesized and we report here its crystal structure.

The asymmetric unit of the title compound comprises of two crystallographically independent molecules, *A* and *B* (Fig. 1). The geometry of both independent molecules indicates a high degree of similarity in terms of their bond distances and bond angles. A comparison of these parameters with some related structures (English *et al.*, 1980) indicates a good agreement. The pyrrole and benzene rings are not coplanar, the dihedral angle being 42.43 (9)° for molecule *A* and 45.70 (9)° for molecule *B*. The crystal packing is stabilized by N—H···O intermolecular hydrogen bonds, generating centrosymmetric dimers (Fig. 2). The crystal structure is further stabilized by weak C—H···O interactions between dimers.

### Experimental

Amide-phosphoryl complex was prepared by treating 1 equiv. of *N*,*N*-dimethyl-4-butoxy benzamide with 3 equiv. of POCl_3_ at room temperature and stirred for 6 h. Subsequently, the reaction product was treated with pyrrole in anhydrous 1,2-dichloroethane at 25°C, stirred for another one hour, and left as such overnight. The resulting mixture was hydrolyzed using saturated sodium carbonate solution, followed by heating for 45 min., to afford the title compound. The compound was extracted with 1,2-dichloroethane and recrystallized from methanol. Single crystals of the compound were obtained by slow evaporation of a methanolic solution.

### Refinement

Atoms H1A and H1B, bonded to N1A and N1B, were located in a difference map and refined freely. Other H atoms were positioned geometrically and were treated as riding on their parent C atoms, with C—H distances of 0.93–0.96 Å, and with *U*_iso_(H) = 1.2–1.5 *U*_eq_(C). The butoxy chains are disordered over two sets of sites with occupancy ratio of 0.701 (7):0.299 (7) and 0.869 (4):0.131 (4), for molecules *A* and *B*, respectively. O—C and C—C bond lengths were restrained to sensible targets values in the disordered parts, and further restraints were applied to displacement parameters of atoms C15A, C15B, C16B, C16D, C17B, C17D, C18B, C18C, and C18D.

### Crystal data

**Table:**

C_15_H_17_NO_2_	*Z* = 4
*M**_r_* = 243.30	*F*(000) = 520
Triclinic, *P*1	*D*_x_ = 1.183 Mg m^−^^3^
Hall symbol: -P 1	Mo *K*α radiation, λ = 0.71073 Å
*a* = 9.4779 (4) Å	Cell parameters from 4854 reflections
*b* = 11.4478 (5) Å	θ = 3.5–29.0°
*c* = 13.1117 (7) Å	µ = 0.08 mm^−^^1^
α = 95.155 (4)°	*T* = 293 K
β = 104.118 (4)°	Block, brown
γ = 94.626 (3)°	0.3 × 0.2 × 0.2 mm
*V* = 1366.31 (11) Å^3^	

### Data collection

**Table:**

Oxford Diffraction Xcalibur Sapphire3 diffractometer	5344 independent reflections
Radiation source: fine-focus sealed tube	3273 reflections with *I* > 2σ(*I*)
Graphite monochromator	*R*_int_ = 0.028
Detector resolution: 16.1049 pixels mm^-1^	θ_max_ = 26.0°, θ_min_ = 3.5°
ω scans	*h* = −11→11
Absorption correction: multi-scan (*CrysAlis RED*; Oxford Diffraction, 2010)	*k* = −14→14
*T*_min_ = 0.935, *T*_max_ = 1.000	*l* = −16→16
13503 measured reflections	

### Refinement

**Table:**

Refinement on *F*^2^	Secondary atom site location: difference Fourier map
Least-squares matrix: full	Hydrogen site location: inferred from neighbouring sites
*R*[*F*^2^ > 2σ(*F*^2^)] = 0.056	H atoms treated by a mixture of independent and constrained refinement
*wR*(*F*^2^) = 0.160	*w* = 1/[σ^2^(*F*_o_^2^) + (0.0667*P*)^2^ + 0.1402*P*] where *P* = (*F*_o_^2^ + 2*F*_c_^2^)/3
*S* = 1.03	(Δ/σ)_max_ = 0.002
5344 reflections	Δρ_max_ = 0.32 e Å^−^^3^
385 parameters	Δρ_min_ = −0.18 e Å^−^^3^
38 restraints	Extinction correction: *SHELXL97* (Sheldrick, 2008), Fc^*^=kFc[1+0.001xFc^2^λ^3^/sin(2θ)]^-1/4^
0 constraints	Extinction coefficient: 0.013 (2)
Primary atom site location: structure-invariant direct methods	

### Fractional atomic coordinates and isotropic or equivalent isotropic displacement parameters (Å^2^)

**Table:**

	*x*	*y*	*z*	*U*_iso_*/*U*_eq_	Occ. (<1)
N1A	0.3964 (2)	0.38234 (18)	0.58381 (17)	0.0676 (5)	
H1A	0.415 (3)	0.456 (2)	0.5625 (19)	0.080 (8)*	
C2A	0.4091 (2)	0.27769 (18)	0.52819 (17)	0.0570 (6)	
C3A	0.3733 (3)	0.1891 (2)	0.58493 (19)	0.0701 (6)	
H3A	0.3731	0.1086	0.5669	0.084*	
C4A	0.3373 (3)	0.2415 (2)	0.6740 (2)	0.0853 (8)	
H4A	0.3086	0.2028	0.7262	0.102*	
C5A	0.3522 (3)	0.3598 (3)	0.6703 (2)	0.0838 (8)	
H5A	0.3344	0.4163	0.7200	0.101*	
C6A	0.4623 (2)	0.27838 (17)	0.43447 (16)	0.0533 (5)	
O7A	0.51771 (17)	0.37123 (12)	0.41197 (12)	0.0678 (4)	
C8A	0.4488 (2)	0.16796 (16)	0.36334 (15)	0.0505 (5)	
C9A	0.3340 (2)	0.08001 (17)	0.34810 (16)	0.0549 (5)	
H9A	0.2654	0.0873	0.3877	0.066*	
C10A	0.3189 (2)	−0.01866 (18)	0.27531 (17)	0.0592 (6)	
H10A	0.2407	−0.0765	0.2658	0.071*	
C11A	0.4209 (3)	−0.03003 (17)	0.21720 (17)	0.0589 (6)	
C12A	0.5375 (3)	0.05661 (18)	0.23217 (18)	0.0626 (6)	
H12A	0.6070	0.0485	0.1935	0.075*	
C13A	0.5504 (2)	0.15421 (17)	0.30394 (17)	0.0579 (6)	
H13A	0.6285	0.2121	0.3129	0.069*	
O14A	0.4161 (2)	−0.12257 (13)	0.14272 (13)	0.0798 (5)	
C15A	0.3187 (7)	−0.2278 (4)	0.1406 (5)	0.0747 (17)	0.701 (7)
H15A	0.2176	−0.2116	0.1176	0.090*	0.701 (7)
H15B	0.3347	−0.2530	0.2108	0.090*	0.701 (7)
C16A	0.3504 (6)	−0.3227 (3)	0.0649 (3)	0.0749 (14)	0.701 (7)
H161	0.4519	−0.3373	0.0894	0.090*	0.701 (7)
H162	0.2902	−0.3948	0.0664	0.090*	0.701 (7)
C17A	0.3236 (6)	−0.2945 (4)	−0.0474 (3)	0.0872 (15)	0.701 (7)
H171	0.3879	−0.2250	−0.0499	0.105*	0.701 (7)
H172	0.2236	−0.2761	−0.0711	0.105*	0.701 (7)
C18A	0.3490 (11)	−0.3948 (5)	−0.1226 (5)	0.0885 (19)	0.701 (7)
H181	0.4461	−0.4167	−0.0973	0.133*	0.701 (7)
H182	0.3381	−0.3698	−0.1918	0.133*	0.701 (7)
H183	0.2789	−0.4614	−0.1261	0.133*	0.701 (7)
C15C	0.2747 (12)	−0.1933 (10)	0.1086 (9)	0.072 (4)	0.299 (7)
H15C	0.1977	−0.1416	0.0956	0.086*	0.299 (7)
H15D	0.2610	−0.2397	0.1645	0.086*	0.299 (7)
C16C	0.2635 (10)	−0.2746 (8)	0.0093 (9)	0.081 (3)	0.299 (7)
H163	0.1744	−0.3280	−0.0049	0.097*	0.299 (7)
H164	0.2563	−0.2280	−0.0497	0.097*	0.299 (7)
C17C	0.3914 (12)	−0.3461 (10)	0.0157 (9)	0.095 (4)	0.299 (7)
H173	0.3998	−0.3924	0.0749	0.114*	0.299 (7)
H174	0.4807	−0.2932	0.0284	0.114*	0.299 (7)
C18C	0.375 (3)	−0.4285 (15)	−0.0856 (10)	0.093 (5)	0.299 (7)
H184	0.2962	−0.4895	−0.0920	0.139*	0.299 (7)
H185	0.4639	−0.4633	−0.0832	0.139*	0.299 (7)
H186	0.3528	−0.3845	−0.1455	0.139*	0.299 (7)
N1B	0.0500 (2)	0.87463 (18)	0.59698 (16)	0.0639 (5)	
H1B	0.042 (3)	0.947 (2)	0.5801 (18)	0.075 (8)*	
C2B	0.0550 (2)	0.78032 (17)	0.52617 (17)	0.0567 (5)	
C3B	0.0572 (3)	0.6824 (2)	0.5801 (2)	0.0712 (7)	
H3B	0.0602	0.6053	0.5523	0.085*	
C4B	0.0540 (3)	0.7193 (2)	0.6838 (2)	0.0781 (7)	
H4B	0.0546	0.6717	0.7377	0.094*	
C5B	0.0497 (3)	0.8383 (2)	0.6909 (2)	0.0716 (7)	
H5B	0.0469	0.8864	0.7513	0.086*	
C6B	0.0488 (2)	0.79522 (18)	0.41681 (18)	0.0586 (6)	
O7B	0.01924 (19)	0.88961 (13)	0.38164 (12)	0.0746 (5)	
C8B	0.0762 (2)	0.69620 (17)	0.34547 (17)	0.0553 (5)	
C9B	0.1814 (2)	0.62104 (18)	0.37939 (17)	0.0618 (6)	
H9B	0.2352	0.6310	0.4497	0.074*	
C10B	0.2078 (2)	0.53160 (18)	0.31047 (18)	0.0627 (6)	
H10B	0.2805	0.4833	0.3343	0.075*	
C11B	0.1270 (2)	0.51385 (17)	0.20688 (17)	0.0547 (5)	
C12B	0.0225 (2)	0.58900 (18)	0.17103 (17)	0.0587 (6)	
H12B	−0.0316	0.5784	0.1008	0.070*	
C13B	−0.0008 (2)	0.67900 (18)	0.23941 (17)	0.0596 (6)	
H13B	−0.0698	0.7298	0.2143	0.072*	
O14B	0.14249 (17)	0.42661 (12)	0.13369 (12)	0.0673 (4)	
C15B	0.2520 (3)	0.3485 (2)	0.1675 (2)	0.0840 (8)	
H15E	0.2263	0.3027	0.2204	0.101*	0.869 (4)
H15F	0.3461	0.3939	0.1988	0.101*	0.869 (4)
H15G	0.2512	0.3191	0.2345	0.101*	0.131 (4)
H15H	0.3497	0.3793	0.1658	0.101*	0.131 (4)
C16B	0.2609 (4)	0.2670 (3)	0.0721 (3)	0.0886 (11)	0.869 (4)
H165	0.2643	0.3134	0.0142	0.106*	0.869 (4)
H166	0.3511	0.2302	0.0894	0.106*	0.869 (4)
C17B	0.1392 (5)	0.1761 (3)	0.0378 (3)	0.1069 (13)	0.869 (4)
H175	0.0510	0.2122	0.0093	0.128*	0.869 (4)
H176	0.1257	0.1384	0.0986	0.128*	0.869 (4)
C18B	0.1608 (6)	0.0812 (5)	−0.0471 (3)	0.127 (2)	0.869 (4)
H18A	0.1743	0.1178	−0.1075	0.191*	0.869 (4)
H18B	0.0760	0.0242	−0.0681	0.191*	0.869 (4)
H18C	0.2454	0.0425	−0.0183	0.191*	0.869 (4)
C16D	0.188 (2)	0.2305 (13)	0.1055 (19)	0.0886 (11)	0.131 (4)
H16A	0.1455	0.1854	0.1521	0.106*	0.131 (4)
H16B	0.1069	0.2457	0.0485	0.106*	0.131 (4)
C17D	0.278 (3)	0.151 (2)	0.0574 (17)	0.1069 (13)	0.131 (4)
H17A	0.2925	0.0863	0.1002	0.128*	0.131 (4)
H17B	0.3732	0.1952	0.0680	0.128*	0.131 (4)
C18D	0.234 (5)	0.097 (4)	−0.057 (2)	0.127 (2)	0.131 (4)
H18D	0.1298	0.0778	−0.0793	0.191*	0.131 (4)
H18E	0.2808	0.0261	−0.0639	0.191*	0.131 (4)
H18F	0.2637	0.1518	−0.1011	0.191*	0.131 (4)

### Atomic displacement parameters (Å^2^)

**Table:**

	*U*^11^	*U*^22^	*U*^33^	*U*^12^	*U*^13^	*U*^23^
N1A	0.0743 (14)	0.0553 (12)	0.0701 (13)	0.0051 (10)	0.0195 (10)	−0.0120 (10)
C2A	0.0581 (13)	0.0514 (12)	0.0563 (13)	0.0084 (10)	0.0085 (10)	−0.0072 (10)
C3A	0.0808 (17)	0.0638 (14)	0.0656 (15)	0.0095 (12)	0.0203 (13)	0.0002 (12)
C4A	0.102 (2)	0.0872 (19)	0.0708 (17)	0.0053 (16)	0.0345 (15)	0.0026 (14)
C5A	0.091 (2)	0.088 (2)	0.0722 (17)	0.0070 (15)	0.0302 (15)	−0.0166 (14)
C6A	0.0511 (13)	0.0462 (12)	0.0567 (13)	0.0074 (10)	0.0042 (10)	−0.0018 (10)
O7A	0.0819 (11)	0.0456 (8)	0.0726 (10)	0.0007 (8)	0.0189 (9)	−0.0024 (7)
C8A	0.0514 (12)	0.0452 (11)	0.0526 (12)	0.0069 (9)	0.0101 (10)	−0.0001 (9)
C9A	0.0508 (13)	0.0577 (12)	0.0553 (13)	0.0039 (10)	0.0163 (10)	−0.0052 (10)
C10A	0.0570 (13)	0.0547 (12)	0.0625 (13)	−0.0083 (10)	0.0182 (11)	−0.0076 (10)
C11A	0.0721 (15)	0.0468 (12)	0.0590 (13)	−0.0006 (10)	0.0257 (11)	−0.0079 (10)
C12A	0.0665 (15)	0.0541 (13)	0.0724 (15)	−0.0009 (11)	0.0339 (12)	−0.0047 (11)
C13A	0.0559 (13)	0.0463 (11)	0.0692 (14)	−0.0043 (9)	0.0176 (11)	−0.0014 (10)
O14A	0.0978 (13)	0.0611 (10)	0.0833 (12)	−0.0177 (9)	0.0482 (10)	−0.0234 (8)
C15A	0.104 (4)	0.046 (3)	0.077 (3)	−0.018 (2)	0.041 (3)	−0.009 (2)
C16A	0.097 (4)	0.056 (2)	0.075 (3)	−0.011 (2)	0.038 (3)	−0.003 (2)
C17A	0.102 (3)	0.088 (3)	0.072 (3)	0.014 (3)	0.029 (3)	−0.011 (2)
C18A	0.117 (5)	0.079 (4)	0.072 (4)	0.006 (3)	0.038 (4)	−0.013 (3)
C15C	0.106 (9)	0.036 (6)	0.073 (8)	−0.012 (5)	0.035 (6)	−0.015 (5)
C16C	0.085 (7)	0.076 (6)	0.079 (8)	0.002 (5)	0.023 (6)	−0.006 (5)
C17C	0.126 (10)	0.104 (8)	0.068 (8)	0.015 (7)	0.049 (7)	0.015 (6)
C18C	0.118 (10)	0.093 (9)	0.076 (9)	0.020 (8)	0.042 (8)	−0.006 (7)
N1B	0.0719 (13)	0.0550 (12)	0.0665 (13)	0.0095 (10)	0.0214 (10)	0.0025 (10)
C2B	0.0596 (14)	0.0480 (12)	0.0604 (14)	0.0006 (10)	0.0154 (11)	−0.0021 (10)
C3B	0.0794 (17)	0.0531 (13)	0.0778 (17)	−0.0040 (11)	0.0190 (13)	0.0020 (12)
C4B	0.0880 (19)	0.0750 (17)	0.0723 (17)	−0.0023 (13)	0.0222 (14)	0.0169 (13)
C5B	0.0720 (17)	0.0831 (18)	0.0608 (15)	0.0056 (13)	0.0219 (12)	0.0027 (13)
C6B	0.0597 (14)	0.0490 (12)	0.0670 (15)	0.0033 (10)	0.0187 (11)	0.0004 (11)
O7B	0.1014 (13)	0.0535 (9)	0.0751 (11)	0.0211 (8)	0.0294 (9)	0.0081 (8)
C8B	0.0596 (13)	0.0458 (11)	0.0596 (13)	0.0052 (10)	0.0153 (11)	0.0017 (10)
C9B	0.0634 (14)	0.0563 (13)	0.0582 (13)	0.0077 (11)	0.0037 (11)	−0.0025 (10)
C10B	0.0622 (14)	0.0540 (13)	0.0683 (15)	0.0132 (11)	0.0089 (12)	0.0030 (11)
C11B	0.0573 (13)	0.0459 (11)	0.0601 (13)	0.0017 (10)	0.0171 (11)	−0.0011 (10)
C12B	0.0609 (14)	0.0594 (13)	0.0549 (13)	0.0080 (11)	0.0127 (11)	0.0053 (10)
C13B	0.0619 (14)	0.0583 (13)	0.0608 (14)	0.0153 (10)	0.0155 (11)	0.0110 (11)
O14B	0.0763 (11)	0.0571 (9)	0.0655 (10)	0.0110 (8)	0.0159 (8)	−0.0061 (7)
C15B	0.0922 (19)	0.0690 (16)	0.0907 (19)	0.0299 (14)	0.0213 (15)	−0.0065 (14)
C16B	0.103 (3)	0.071 (2)	0.101 (3)	0.0203 (19)	0.042 (2)	0.0037 (17)
C17B	0.142 (4)	0.082 (2)	0.089 (3)	0.009 (2)	0.023 (2)	−0.0127 (19)
C18B	0.209 (6)	0.080 (3)	0.090 (2)	0.008 (3)	0.045 (3)	−0.016 (2)
C16D	0.103 (3)	0.071 (2)	0.101 (3)	0.0203 (19)	0.042 (2)	0.0037 (17)
C17D	0.142 (4)	0.082 (2)	0.089 (3)	0.009 (2)	0.023 (2)	−0.0127 (19)
C18D	0.209 (6)	0.080 (3)	0.090 (2)	0.008 (3)	0.045 (3)	−0.016 (2)

### Geometric parameters (Å, º)

**Table:**

N1A—C5A	1.340 (3)	N1B—C5B	1.335 (3)
N1A—C2A	1.375 (3)	N1B—C2B	1.371 (3)
N1A—H1A	0.92 (2)	N1B—H1B	0.88 (2)
C2A—C3A	1.379 (3)	C2B—C3B	1.377 (3)
C2A—C6A	1.438 (3)	C2B—C6B	1.447 (3)
C3A—C4A	1.394 (3)	C3B—C4B	1.395 (3)
C3A—H3A	0.9300	C3B—H3B	0.9300
C4A—C5A	1.356 (4)	C4B—C5B	1.362 (3)
C4A—H4A	0.9300	C4B—H4B	0.9300
C5A—H5A	0.9300	C5B—H5B	0.9300
C6A—O7A	1.239 (2)	C6B—O7B	1.239 (2)
C6A—C8A	1.479 (3)	C6B—C8B	1.484 (3)
C8A—C9A	1.385 (3)	C8B—C9B	1.387 (3)
C8A—C13A	1.388 (3)	C8B—C13B	1.392 (3)
C9A—C10A	1.386 (3)	C9B—C10B	1.383 (3)
C9A—H9A	0.9300	C9B—H9B	0.9300
C10A—C11A	1.376 (3)	C10B—C11B	1.375 (3)
C10A—H10A	0.9300	C10B—H10B	0.9300
C11A—O14A	1.365 (2)	C11B—O14B	1.364 (2)
C11A—C12A	1.388 (3)	C11B—C12B	1.387 (3)
C12A—C13A	1.372 (3)	C12B—C13B	1.372 (3)
C12A—H12A	0.9300	C12B—H12B	0.9300
C13A—H13A	0.9300	C13B—H13B	0.9300
O14A—C15A	1.451 (5)	O14B—C15B	1.439 (3)
O14A—C15C	1.456 (9)	C15B—C16D	1.512 (10)
C15A—C16A	1.503 (6)	C15B—C16B	1.515 (3)
C15A—H15A	0.9700	C15B—H15E	0.9700
C15A—H15B	0.9700	C15B—H15F	0.9700
C16A—C17A	1.501 (5)	C15B—H15G	0.9700
C16A—H161	0.9700	C15B—H15H	0.9700
C16A—H162	0.9700	C16B—C17B	1.444 (5)
C17A—C18A	1.518 (6)	C16B—H165	0.9700
C17A—H171	0.9700	C16B—H166	0.9700
C17A—H172	0.9700	C17B—C18B	1.546 (5)
C18A—H181	0.9600	C17B—H175	0.9700
C18A—H182	0.9600	C17B—H176	0.9700
C18A—H183	0.9600	C18B—H18A	0.9600
C15C—C16C	1.507 (9)	C18B—H18B	0.9600
C15C—H15C	0.9700	C18B—H18C	0.9600
C15C—H15D	0.9700	C16D—C17D	1.496 (10)
C16C—C17C	1.506 (9)	C16D—H16A	0.9700
C16C—H163	0.9700	C16D—H16B	0.9700
C16C—H164	0.9700	C17D—C18D	1.519 (10)
C17C—C18C	1.525 (9)	C17D—H17A	0.9700
C17C—H173	0.9700	C17D—H17B	0.9700
C17C—H174	0.9700	C18D—H18D	0.9600
C18C—H184	0.9600	C18D—H18E	0.9600
C18C—H185	0.9600	C18D—H18F	0.9600
C18C—H186	0.9600		
			
C5A—N1A—C2A	109.3 (2)	C17C—C18C—H186	109.5
C5A—N1A—H1A	126.9 (15)	H184—C18C—H186	109.5
C2A—N1A—H1A	123.8 (15)	H185—C18C—H186	109.5
N1A—C2A—C3A	106.6 (2)	C5B—N1B—C2B	109.9 (2)
N1A—C2A—C6A	120.1 (2)	C5B—N1B—H1B	126.4 (15)
C3A—C2A—C6A	133.22 (19)	C2B—N1B—H1B	123.6 (15)
C2A—C3A—C4A	107.9 (2)	N1B—C2B—C3B	106.4 (2)
C2A—C3A—H3A	126.1	N1B—C2B—C6B	120.85 (18)
C4A—C3A—H3A	126.1	C3B—C2B—C6B	132.7 (2)
C5A—C4A—C3A	107.1 (2)	C2B—C3B—C4B	108.0 (2)
C5A—C4A—H4A	126.5	C2B—C3B—H3B	126.0
C3A—C4A—H4A	126.5	C4B—C3B—H3B	126.0
N1A—C5A—C4A	109.2 (2)	C5B—C4B—C3B	106.9 (2)
N1A—C5A—H5A	125.4	C5B—C4B—H4B	126.5
C4A—C5A—H5A	125.4	C3B—C4B—H4B	126.5
O7A—C6A—C2A	120.48 (18)	N1B—C5B—C4B	108.8 (2)
O7A—C6A—C8A	119.59 (19)	N1B—C5B—H5B	125.6
C2A—C6A—C8A	119.92 (19)	C4B—C5B—H5B	125.6
C9A—C8A—C13A	117.99 (18)	O7B—C6B—C2B	120.60 (19)
C9A—C8A—C6A	122.99 (18)	O7B—C6B—C8B	119.5 (2)
C13A—C8A—C6A	118.90 (19)	C2B—C6B—C8B	119.88 (18)
C8A—C9A—C10A	121.60 (19)	C9B—C8B—C13B	117.58 (19)
C8A—C9A—H9A	119.2	C9B—C8B—C6B	122.3 (2)
C10A—C9A—H9A	119.2	C13B—C8B—C6B	120.08 (18)
C11A—C10A—C9A	119.27 (19)	C10B—C9B—C8B	121.1 (2)
C11A—C10A—H10A	120.4	C10B—C9B—H9B	119.4
C9A—C10A—H10A	120.4	C8B—C9B—H9B	119.4
O14A—C11A—C10A	124.27 (19)	C11B—C10B—C9B	120.23 (19)
O14A—C11A—C12A	115.79 (18)	C11B—C10B—H10B	119.9
C10A—C11A—C12A	119.94 (19)	C9B—C10B—H10B	119.9
C13A—C12A—C11A	120.14 (19)	O14B—C11B—C10B	124.59 (18)
C13A—C12A—H12A	119.9	O14B—C11B—C12B	115.90 (19)
C11A—C12A—H12A	119.9	C10B—C11B—C12B	119.50 (19)
C12A—C13A—C8A	121.06 (19)	C13B—C12B—C11B	119.8 (2)
C12A—C13A—H13A	119.5	C13B—C12B—H12B	120.1
C8A—C13A—H13A	119.5	C11B—C12B—H12B	120.1
C11A—O14A—C15A	118.1 (3)	C12B—C13B—C8B	121.65 (19)
C11A—O14A—C15C	113.8 (5)	C12B—C13B—H13B	119.2
O14A—C15A—C16A	108.0 (4)	C8B—C13B—H13B	119.2
O14A—C15A—H15A	110.1	C11B—O14B—C15B	117.42 (17)
C16A—C15A—H15A	110.1	O14B—C15B—C16D	104.5 (9)
O14A—C15A—H15B	110.1	O14B—C15B—C16B	108.6 (2)
C16A—C15A—H15B	110.1	O14B—C15B—H15E	110.0
H15A—C15A—H15B	108.4	C16B—C15B—H15E	110.0
C17A—C16A—C15A	114.5 (5)	O14B—C15B—H15F	110.0
C17A—C16A—H161	108.6	C16B—C15B—H15F	110.0
C15A—C16A—H161	108.6	H15E—C15B—H15F	108.4
C17A—C16A—H162	108.6	O14B—C15B—H15G	114.2
C15A—C16A—H162	108.6	C16B—C15B—H15G	121.5
H161—C16A—H162	107.6	O14B—C15B—H15H	113.8
C16A—C17A—C18A	113.1 (5)	C16D—C15B—H15H	118.5
C16A—C17A—H171	109.0	H15E—C15B—H15H	126.6
C18A—C17A—H171	109.0	H15G—C15B—H15H	111.7
C16A—C17A—H172	109.0	C17B—C16B—C15B	113.1 (3)
C18A—C17A—H172	109.0	C17B—C16B—H165	109.0
H171—C17A—H172	107.8	C15B—C16B—H165	109.0
O14A—C15C—C16C	111.5 (8)	C17B—C16B—H166	109.0
O14A—C15C—H15C	109.3	C15B—C16B—H166	109.0
C16C—C15C—H15C	109.3	H165—C16B—H166	107.8
O14A—C15C—H15D	109.3	C16B—C17B—C18B	113.0 (3)
C16C—C15C—H15D	109.3	C16B—C17B—H175	109.0
H15C—C15C—H15D	108.0	C18B—C17B—H175	109.0
C17C—C16C—C15C	113.5 (10)	C16B—C17B—H176	109.0
C17C—C16C—H163	108.9	C18B—C17B—H176	109.0
C15C—C16C—H163	108.9	H175—C17B—H176	107.8
C17C—C16C—H164	108.9	C17D—C16D—C15B	122.0 (15)
C15C—C16C—H164	108.9	C17D—C16D—H16A	106.8
H163—C16C—H164	107.7	C15B—C16D—H16A	106.8
C16C—C17C—C18C	111.8 (12)	C17D—C16D—H16B	106.8
C16C—C17C—H173	109.2	C15B—C16D—H16B	106.8
C18C—C17C—H173	109.2	H16A—C16D—H16B	106.7
C16C—C17C—H174	109.2	C16D—C17D—C18D	124 (3)
C18C—C17C—H174	109.3	C16D—C17D—H17A	106.4
H173—C17C—H174	107.9	C18D—C17D—H17A	106.4
C17C—C18C—H184	109.5	C16D—C17D—H17B	106.4
C17C—C18C—H185	109.5	C18D—C17D—H17B	106.4
H184—C18C—H185	109.5	H17A—C17D—H17B	106.5
			
C5A—N1A—C2A—C3A	−1.0 (3)	C15C—C16C—C17C—C18C	179.1 (11)
C5A—N1A—C2A—C6A	−176.9 (2)	C5B—N1B—C2B—C3B	0.2 (3)
N1A—C2A—C3A—C4A	0.7 (3)	C5B—N1B—C2B—C6B	176.8 (2)
C6A—C2A—C3A—C4A	175.8 (2)	N1B—C2B—C3B—C4B	−0.2 (3)
C2A—C3A—C4A—C5A	−0.2 (3)	C6B—C2B—C3B—C4B	−176.2 (2)
C2A—N1A—C5A—C4A	0.9 (3)	C2B—C3B—C4B—C5B	0.1 (3)
C3A—C4A—C5A—N1A	−0.4 (3)	C2B—N1B—C5B—C4B	−0.2 (3)
N1A—C2A—C6A—O7A	11.1 (3)	C3B—C4B—C5B—N1B	0.0 (3)
C3A—C2A—C6A—O7A	−163.5 (2)	N1B—C2B—C6B—O7B	−9.8 (3)
N1A—C2A—C6A—C8A	−168.00 (18)	C3B—C2B—C6B—O7B	165.7 (2)
C3A—C2A—C6A—C8A	17.5 (4)	N1B—C2B—C6B—C8B	171.02 (18)
O7A—C6A—C8A—C9A	−146.7 (2)	C3B—C2B—C6B—C8B	−13.4 (4)
C2A—C6A—C8A—C9A	32.4 (3)	O7B—C6B—C8B—C9B	142.7 (2)
O7A—C6A—C8A—C13A	29.2 (3)	C2B—C6B—C8B—C9B	−38.1 (3)
C2A—C6A—C8A—C13A	−151.68 (19)	O7B—C6B—C8B—C13B	−34.7 (3)
C13A—C8A—C9A—C10A	−0.6 (3)	C2B—C6B—C8B—C13B	144.5 (2)
C6A—C8A—C9A—C10A	175.36 (19)	C13B—C8B—C9B—C10B	−0.6 (3)
C8A—C9A—C10A—C11A	0.4 (3)	C6B—C8B—C9B—C10B	−178.0 (2)
C9A—C10A—C11A—O14A	−179.3 (2)	C8B—C9B—C10B—C11B	−1.6 (3)
C9A—C10A—C11A—C12A	0.2 (3)	C9B—C10B—C11B—O14B	−178.45 (19)
O14A—C11A—C12A—C13A	178.81 (19)	C9B—C10B—C11B—C12B	2.4 (3)
C10A—C11A—C12A—C13A	−0.7 (3)	O14B—C11B—C12B—C13B	179.75 (18)
C11A—C12A—C13A—C8A	0.5 (3)	C10B—C11B—C12B—C13B	−1.0 (3)
C9A—C8A—C13A—C12A	0.1 (3)	C11B—C12B—C13B—C8B	−1.2 (3)
C6A—C8A—C13A—C12A	−176.02 (19)	C9B—C8B—C13B—C12B	2.0 (3)
C10A—C11A—O14A—C15A	−14.7 (4)	C6B—C8B—C13B—C12B	179.5 (2)
C12A—C11A—O14A—C15A	165.8 (4)	C10B—C11B—O14B—C15B	−0.5 (3)
C10A—C11A—O14A—C15C	15.6 (6)	C12B—C11B—O14B—C15B	178.7 (2)
C12A—C11A—O14A—C15C	−163.9 (6)	C11B—O14B—C15B—C16D	145.8 (10)
C11A—O14A—C15A—C16A	−171.0 (3)	C11B—O14B—C15B—C16B	−174.4 (2)
C15C—O14A—C15A—C16A	101.0 (15)	O14B—C15B—C16B—C17B	−74.7 (3)
O14A—C15A—C16A—C17A	−62.0 (7)	C16D—C15B—C16B—C17B	15.3 (15)
C15A—C16A—C17A—C18A	−177.0 (5)	C15B—C16B—C17B—C18B	−170.5 (3)
C11A—O14A—C15C—C16C	166.2 (7)	O14B—C15B—C16D—C17D	139 (2)
C15A—O14A—C15C—C16C	−88.3 (16)	C16B—C15B—C16D—C17D	36.7 (15)
O14A—C15C—C16C—C17C	50.3 (14)	C15B—C16D—C17D—C18D	−130 (3)

### Hydrogen-bond geometry (Å, º)

**Table:**

*D*—H···*A*	*D*—H	H···*A*	*D*···*A*	*D*—H···*A*
N1*A*—H1*A*···O7*A*^i^	0.93 (3)	2.00 (2)	2.864 (2)	154 (2)
N1*B*—H1*B*···O7*B*^ii^	0.88 (3)	2.06 (2)	2.834 (3)	146 (2)
C9*B*—H9*B*···O7*A*^i^	0.93	2.59	3.426 (3)	149

Symmetry codes: (i) −*x*+1, −*y*+1, −*z*+1; (ii) −*x*, −*y*+2, −*z*+1.
